# The many faceted role of glycogen synthase kinase-3 (GSK-3) in T cells and cancer immunotherapy

**DOI:** 10.46439/cancerbiology.5.058

**Published:** 2024

**Authors:** Aurora Rivas Crespo, Silvia Guil Luna, Bastien Moës, Antonio Rodriguez, Christopher E Rudd

**Affiliations:** 1 Maimonides Biomedical Research Institute of Córdoba (IMIBIC), Córdoba, Spain; 2 Cancer Network Biomedical Research Centre (CIBERONC), Madrid, Spain; 3 Andalusia-ROCHE Network Mixed Alliance in Precision Medical Oncology, Spain; 4 Division of Immunology-Oncology, Centre de Recherche Hôpital Maisonneuve-Rosemont (CR-HMR), Montreal, QC, Canada; 5 Faculty of Medicine, Université de Montreal, Montreal, Canada; 6 Department of Microbiology, Infection and Immunology, Faculty of Medicine, Université de Montreal, Montreal, QC, Canada

## Introduction

Originally identified for its involvement in phosphorylating glycogen synthase and regulating glucose metabolism in response to insulin, glycogen synthase kinase-3 (GSK-3) has since been recognized as a versatile serine/threonine kinase with diverse functions [1,2]. Extensive research has demonstrated that GSK-3 phosphorylates over 100 protein substrates where it intersects numerous signaling pathways. While it was initially implicated in the regulation of glucose metabolism, subsequent investigations revealed an impact of GSK-3 in cellular processes beyond glycogen synthase phosphorylation and glycogen metabolism [3]. This kinase regulates key cellular functions from signaling to metabolism, proliferation, and differentiation and has been implicated in many pathophysiological conditions, including neurodegenerative disorders, cancer, diabetes, inflammation, and mood disorders.

GSK-3 is encoded by two distinct genes located on chromosomes 19 and 3, giving rise to two unique isoforms: GSK-3α (51 kDa) and GSK-3β (47 kDa). These isoforms share a 97% amino acid sequence identity within their catalytic domains and an overall amino acid sequence similarity of 84%. This similarity suggests that they likely to perform overlapping biological functions even with the only presence in GSK-3α of a glycine rich domain in its N-terminal extremity [4]. Phosphorylation at residue Ser21 and Ser9by serine kinases inactivates GSK-3α andGSK-3β respectively, limiting its ability to phosphorylate downstream targets [5]. On the other hand, tyrosine kinase-mediated phosphorylation at Tyr279 andTyr216 enhances the catalytic activity of GSK-3α and GSK-3β respectively, augmenting their capacity to phosphorylate specific substrates. This dual regulatory mechanism provides GSK-3α and GSK-3β with a means to fine tune its activity, depending on the cellular context and signaling cues. The balance between phosphorylation at Ser21/9 and Tyr279/216 serves as a crucial molecular switch controlling GSK-3β’s activity and its participation in diverse cellular functions.

Of key interest to both immune function and cancer growth, in contrast to other kinases, GSK-3 maintains a state of constitutive activity in resting cells, playing a role in keeping cells quiescent. It therefore can be viewed as a checkpoint in limiting the proliferation of cells and in the case of T-cells, limiting their ability to respond to antigen and to cancer neoantigens.

### Signaling Pathway

**Figure 1.**
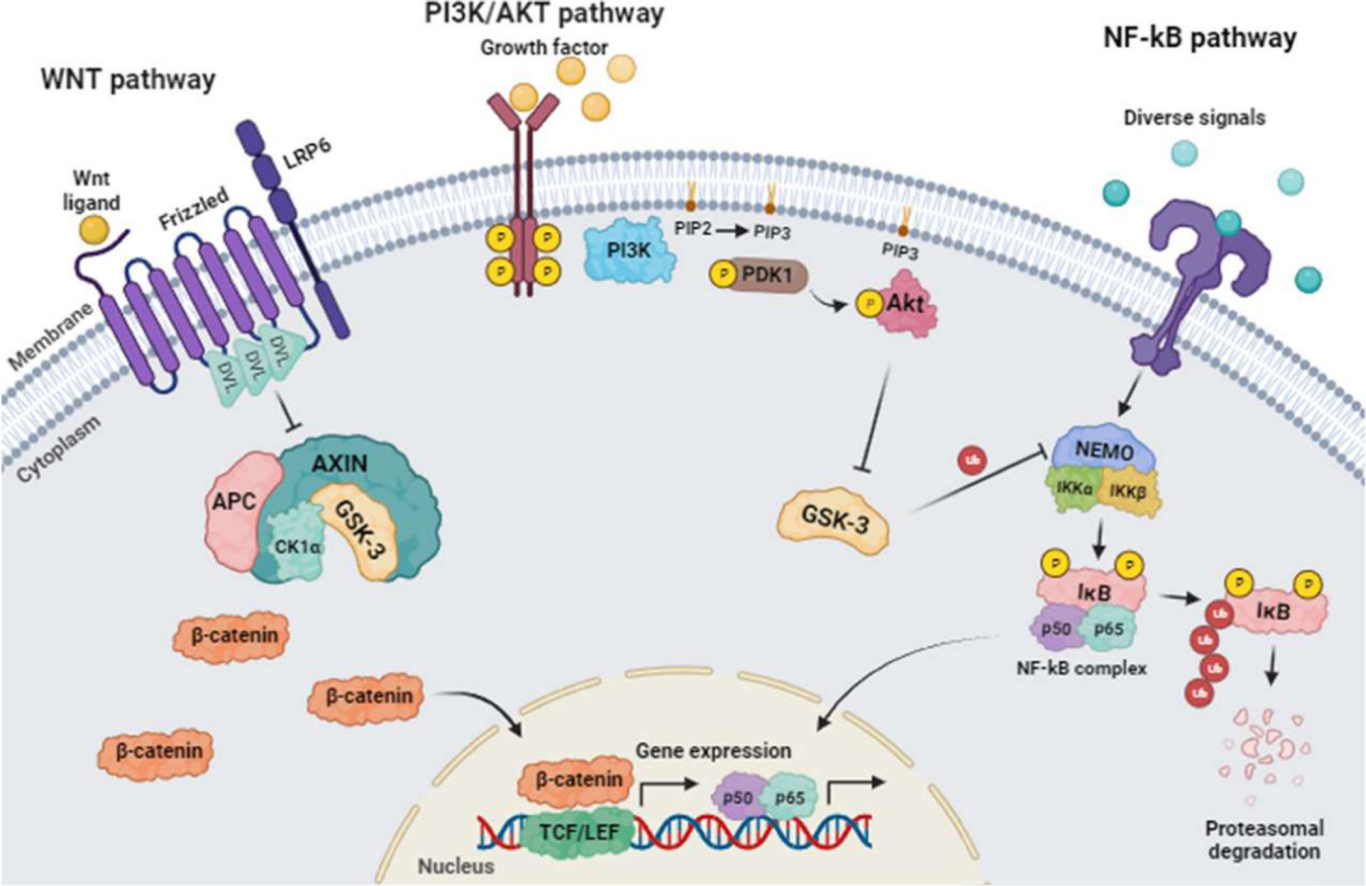
Overview of the main signaling pathways in which GSK-3 is involved. When Wnt ligands activate the Wnt pathway, it inhibits the complex formed by GSK-3, APC, Axin, and CK1α by activating Dvl. This results in the cytosolic accumulation of beta-catenin, which then translocate into the nucleus and interacts with TCF/LEF to induce gene expression. Second, the activation of the PI3K/AKT pathway is triggered by growth factors or other signals. This activation increases the catalytic activity of PI3K and brings it closer to its substrate PIP2. As a result, PIP2 is converted to PIP3. PIP3 then recruits and activates PDK-1, which further activates AKT. The last step involves the inhibition of GSK-3 caused by the stimulation of AKT. Finally, various signals can activate the IKK complex in the NF-Kb pathway. This complex consists of the regulatory subunit NEMO/IKKγ and two catalytic subunits, IKKα and IKKβ. GSK-3 can phosphorylate the NEMO regulatory subunit, which stabilizes it and prevents the two catalytic subunits from phosphorylating the inhibitory protein IkB. This, in turn, stops the proteasomal degradation of IkB and the translocation of the p50 and p65 subunits to the nucleus. As a result, gene expression is not induced.

Perhaps the best-known GSK-3 linked pathway involves Wnt/β-catenin signaling [6,7] (Figure 1). In this pathway, GSK-3 is in a multiprotein complex formed by axin, β-catenin, and the adenomatous polyposis coli (APC) protein. Under unstimulated conditions, GSK-3 is active and bound to this complex, where it can phosphorylate β-catenin leading to ubiquitin-mediated proteolytic destruction. When the Wnt pathway is activated, Dvl is activated, resulting in the inhibition of GSK-3, so that β-catenin is not phosphorylated. This leads to the accumulation of β-catenin in the cytosol and its translocation to the nucleus where it interacts with the transcription factor LCF/TCF (lymphoid enhancer factor/T cell factor) for the expression of different target genes [8–10].

Another signaling pathway in multiple cellular functions is lipid and serine kinase phosphatidylinositol 3-kinase (PI-3K) and the serine/threonine kinase protein kinase B (PKB) or AKT (Figure 1). Various signals can activate PI-3K, which stimulates the generation phosphatidylinositol (3,4,5)-trisphosphate (PIP3). The lipid kinase function of class I PI3Ks is carried out by the p110 catalytic subunit, which consists of four isoforms divided into class IA, namely p110α, p110β, and p110δ. The p110γ isoform, belonging to class IB, encoded by the PIK3CG gene, plays a pivotal role as a central controller of immune cell activity [11]. PIP3 recruits and activates different proteins, including AKT/PKB, via their pleckstrin-homology (PH) domains which binds to PIP3. AKT can then phosphorylate GSK-3 leading to its inhibition. GSK-3 inhibition in turn has phosphorylates multiple substrates such as c-myc, c-jun, SNAIL, or HIF1α [10,12].

In another context, GSK-3 is also involved in the signaling pathway of the proinflammatory transcription factor NF-κB (Figure 1). In cellular resting situations, NF-κB is inactive in the cytoplasm associated with IKKB inhibitory proteins. However, stimulation of this pathway results in activation of the IκB kinase (IKK) complex, consisting of the NF-κB Essential Modifier regulatory subunit (NEMO/IKKγ) and two catalytic subunits (IKKα and IKKβ), which phosphorylate NF-κB inhibitory IκB proteins [13]. Phosphorylation of IκB leads to its proteasomal degradation, resulting in the release of NF-κB, its translocation to the nucleus, and the transcriptional regulation of different genes related to inflammation and survival, among others. GSK-3 regulates this NEMO protein; therefore, inhibiting GSK-3 leads to the destabilization of NEMO, its proteasomal degradation, and decreased NF-κB activity [14]. Thus, inhibition of GSK-3 leads to a reduction of NF-κB-regulated gene expression.

### GSK-3 in Immune Cells

Efficient elimination of tumors relies on the activation of immune T-cells, which involves the interaction of the T-cell receptor (TCR) and coreceptors, CD4 and CD8. Our previous studies showed that these coreceptors bind to the protein-tyrosine kinase p56^lck^ (LCK), initiating a cascade of protein tyrosine phosphorylation [15,16]. LCK phosphorylates tyrosine residues within the immunoreceptor tyrosine-based activation motifs (ITAMs) of the ζ chains present in the TCR complex [17]. This phosphorylation event creates specific docking sites for another protein tyrosine kinase called ZAP-70 [18,19]. ZAP-70 and p56^lck^ then phosphorylate downstream immune cell adaptor proteins, namely the linker of activated T-cells (LAT) and the SH2-domain containing signal transducing adaptor protein (SLP-76) [17]. Phosphorylated LAT serves as a docking site for SLP-76, ultimately leading to the activation, proliferation, differentiation, and cytokine release of T-cells [20]. Broadly speaking, Src kinases like p56^lck^ have the capability to phosphorylate a wide range of substrates, while ZAP-70 exhibits a more limited number of targets [21].

In contrast, GSK-3 is constitutively active in resting T-cells [4]. It trans-locates to the nucleus of CD4+ T-cells to facilitate the exit of NFAT through its phosphorylation, while active GSK-3β inhibits T-cell proliferation [22,23]. While signals from the T-cell receptor (TCR) and CD28 can inactivate GSK-3 in T-cells, CD28 binds to and activates PI-3K leading to the phosphorylation of phosphatidylinositol-(4,5)-biphosphate (PIP2), which is subsequently converted to phosphatidylinositol-(3,4,5)- triphosphate (PIP3) at the plasma membrane [24]. These lipid molecules in turn recruit proteins containing (PH) domains. Pyruvate dehydrogenase kinase 1 (PDK1), in conjunction with an unidentified kinase, phosphorylates at two specific sites (Thr308 and Ser473) and activates PKB or AKT, which carry PH domains. PKB, along with other kinases, then inactivates GSK-3β and GSK-3α by phosphorylating inhibitory residues 9 and 21 [56].

Upon activation of the T-cell receptor (TCR), CD8+ T-cells undergo differentiation into memory, memory-effector, and effector cytolytic T-cells, while CD4+ T-cells differentiate into various helper T-cells or suppressive Tregs [13,14]. Effector T-cells undergo expansion, efficiently eliminating pathogens, and later undergo apoptosis or differentiate into memory T-cells, ensuring enduring immune defense upon subsequent encounters with the same pathogens [15,16]. Nevertheless, in prolonged conditions like cancer or chronic infection, continuous stimulation can hinder the functionality of T-cells, resulting in a state of dysfunction referred to as T-cell exhaustion [17]. This state is characterized by a gradual loss of T-cell effector function. Following TCR activation in context of chronic infection or cancer, inhibitory receptors (IRs) such as programmed cell death protein 1 (PD-1), lymphocyte activation gene 3 (LAG-3), cytotoxic T lymphocyte-associated protein 4 (CTLA-4) T cell immunoglobulin and mucin domain-containing protein 3 (TIM3) and CD101 are upregulated [18,19]. These immune receptors (IRs) act as indicators of exhausted T-cells and exert diverse levels of control over T-cell exhaustion. In the realms of cancer biology and chronic infection models, the inhibition of these receptors, commonly referred to as immune checkpoint blockade (ICB), can partially reverse T-cell exhaustion. This reversal empowers T-cells to multiply and generate a vigorous immune response against cancer or the infectious agent [1,20]. Notably, in the case of cancer, the administration of antibodies targeting CTLA-4, followed by antibodies targeting PD-1 or a combination of both, has demonstrated effectiveness in reducing tumor growth [1,21].

**Figure 2.**
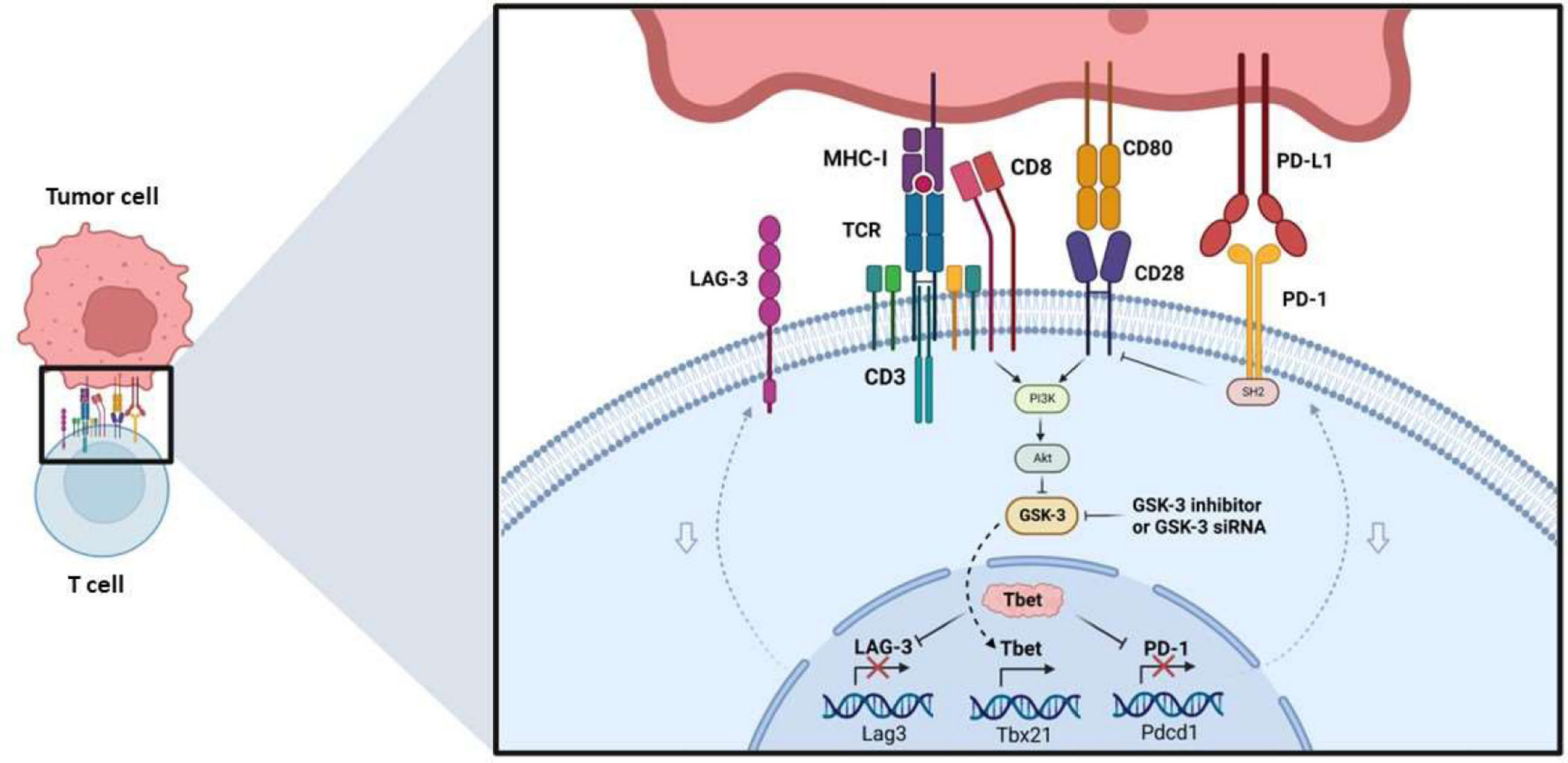
Role of GSK-3 in antitumor immunity. Activation of the TCR complex and CD28 results in the inhibition of GSK-3 by stimulating the PI3K/AKT pathway. The PD-1 signaling specifically recruits the phosphatase Shp2, which dephosphorylates CD28 and blocks its signaling. Consequently, when this kinase is inhibited by small molecule inhibitors (SMI) or siRNA, it leads to increased expression of TBET (Tbx21). Tbet is a transcriptional inhibitor of PD-1 and LAG-3, resulting in decreased expression of both inhibitory receptors. This leads to increased cytotoxic activity against tumor cells.

The control of PD-1 and LAG-3 expression by glycogen synthase kinase 3 (GSK-3) presents an intriguing aspect of research (Figure 2). Our studies have demonstrated the central role of GSK-3 in regulating PD-1 and LAG-3 in T-cells [25–27]. Using small molecule inhibitors (SMIs) or siRNA to downregulate expression or inhibit GSK-3 activity (GSK-3i), we observed a significant increase in the cytolytic activity of CD8+ T-cells. This enhancement in CD8+ T-cell function was primarily attributed to the reduction in PD-1 expression following GSK-3 inhibition, and later we observed a similar effect on LAG-3 as well. In mouse models of tumor growth, we found that the use of GSK-3 inhibitors such as SB415286 resulted in a reduction in tumor growth comparable to the effects of anti-PD-1 therapy in both B16 melanoma and EL4 lymphoma models [58]. The pre-treatment of T-cells with SMIs prior to adaptive cell therapy also potentiated the regression tumor growth by activated T-cells [25–27]. Furthermore, GSK-3i showed comparable efficacy to anti-PD-1 in promoting the clearance of lymphocytic choriomeningitis virus clone 13 [26]. Combining the inhibition of LAG-3 with GSK-3 inhibition further suppressed tumor growth and significantly prolonged the survival of mice, surpassing the outcomes of single-agent therapy.

Mechanistically, the impact of GSK-3 inhibition is partially mediated by an increase in the expression of the transcriptional regulator TBET (Tbx21) [26]. TBET, in turn, binds to and inhibits the transcription of PD-1 and LAG-3 [25]. Additionally, GSK-3 inactivation compensates for the absence of CD28 in the priming of CD8+ cytotoxic T-cells [28]. At the cellular level, GSK-3 inhibition led to decreased T-cell motility and reduced interactions between CD8+ T-cells and target cells, while maintaining their cytolytic activity [29]. Overall, the regulation of immune checkpoint receptor (IR) expression by GSK-3 presents a promising pathway to explore for the development of novel strategies in cancer treatment.

### GSK-3 in Tumors

On the other side, GSK-3 is involved in multiple pathways and also regulates critical cell functions commonly altered in cancer, such as proliferation, metabolism, cell survival, and apoptosis [30]. This suggests that GSK-3 may play a relevant role in cancer. Intriguingly, there is ongoing discussion and conflicting perspectives surrounding the role that GSK-3 plays in cancer. On the one hand, considering the regulation of certain pathways such as Wnt/beta-catenin, GSK-3 could exert an anti-tumorigenic role, and thus, its inhibition would promote the proliferation of the tumor cells [31]. However, regulating other pathways, such as TNF/NF-κB or STAT3/NFATC2 axis, would support the hypothesis that GSK-3 exerts a pro-tumorigenic role in some cancer by promoting a sustained inflammatory state [32,33].

Nevertheless, GSK-3 deregulation has been observed in various types of cancer. Compared to respective healthy cells and tissue, this kinase is overexpressed and/or highly activated in the tumor tissue in several cancers-including glioblastoma, osteosarcoma, colon, bladder, renal, or gastrointestinal [34–39]. In various types of cancers, such as glioblastoma, osteosarcoma, colon, bladder, renal, or gastrointestinal cancers, this kinase is found to be significantly upregulated and/or highly activated in tumor tissue when compared to corresponding healthy cells and tissues. GSK-3 upregulation was found to promote proliferation, migration, or invasion of cancer cells notably by over-activating HIF1α/VEGFA signaling and influencing cytoskeleton formation [40,41]. In this context, the expression of GSK-3 has been associated with worse survival or poor prognostic factors in patients with lung, ovarian, or colon cancer, among others [42–44]. These findings suggest that GSK-3 may promote cancer development and its hallmarks.

In this sense, some studies maintain that GSK-3 promotes the intrinsic characteristics of cancer. It has been reported that inhibiting this kinase reduces tumor cell survival and proliferation, leading to cell death in various types of cancer. Different mechanisms causing this antitumor effect has been described, including the reduction in the expression of pro-survival genes through the NF-κB pathway or Rb-mediated cell cycle regulation [35,37,45]. In addition, GSK-3 inhibition can overcome apoptosis resistance through various mechanisms, such as sensitizing TRAIL-induced apoptosis or inducing p53-dependent apoptosis via Bax [46,47].

One key event linked to cancer is that it spread via metastatic mechanisms. In fact, the metastatic spread of cancer cells, which begins with local migration and invasion, is a leading cause of cancer-related death. In this context, as we also observed with immune cells, GSK-3 inhibition also attenuates cell migration and invasion in certain types of cancer, such as pancreatic cancer or glioblastoma [48,49]. On the other hand, it has also been suggested that inhibiting GSK-3β can increase the migratory and invasive capacity of nasopharyngeal carcinoma [50]. In this context, high-grade (BD3) TB CRC is associated with elevated expression levels of GSK-3β isoform, together with an increased expression of PD-L1 in tumor cells [51]. This emphasizes the need for further research into the role of GSK-3 in various cancer hallmarks, which may depend on cancer type.

In addition, the inhibition of this kinase can also overcome resistance to chemotherapy or radiotherapy in different cancers, such as breast, pancreatic, glioblastoma or colon cancer [52–55]. Interestingly, WNT4, a protein secreted by tumor tissues in colorectal cancer, activates WNT/β-catenin signaling leading to GSK-3 inactivation [56]. Furthermore, GSK-3 inhibition has been shown to enhance the effects of chemotherapeutic agents in other studies [57].

## Conclusion

**Figure 3.**
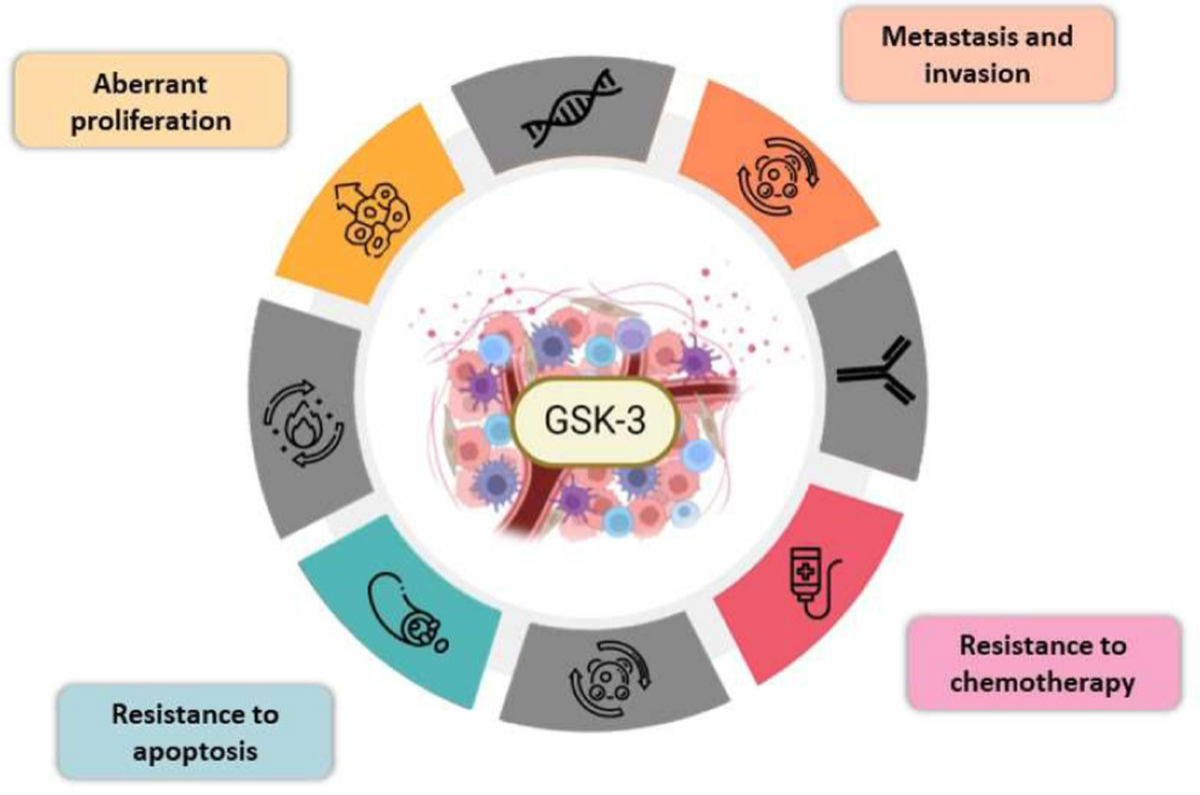
Involvement of GSK-3 in different hallmarks of cancer. Illustration of the hallmarks of cancer with GSK-3 at the center, highlighting its role in the malignancy and the four following hallmarks: abnormal proliferation, invasion and metastasis, resistance to chemotherapy, and apoptosis.

GSK-3 plays a pleiotropic role in regulating both cancer growth and the development of an immune response against tumor antigens (Figure 3). The balance between these events will be key in determining how this pathway can be manipulated in the therapeutic approaches to treating cancer. Depending on the cancer type, the inhibition of GSK-3 may both directly inhibit tumor growth while concurrently promoting immune cell rejection of the tumor. Due to these findings, we and others have proposed that the inhibition of GSK-3 as a potential therapy for multiple cancer types and/or as an additional therapy to enhance current treatments. However, more in-depth research is needed to explore the efficacy of this therapy on different types of cancer and its impact on the various cancer hallmarks.
